# Association between intimate partner violence and nutritional status among Indian women: a latent class analysis approach

**DOI:** 10.1186/s13690-023-01152-w

**Published:** 2023-08-29

**Authors:** Pravat Bhandari, Ranjan Kumar Prusty, Shahina Begum

**Affiliations:** 1 Department of Biostatistics, ICMR-National Institute for Research in Reproductive and Child Health, Mumbai, India; 2 International Institute for Population Sciences (IIPS), Mumbai, India

**Keywords:** Intimate partner violence, Domestic violence, Undernutrition, Latent class analysis, India

## Abstract

**Background:**

Intimate partner violence (IPV) is an unabating public health issue that has numerous negative repercussions for women’s health. Its detrimental impact on women’s nutritional outcomes has been documented in a few studies from low- and middle-income countries; however, there is a lack of granular understanding in terms of the typology of IPV experiences and their association with nutritional outcomes. This study explores the distinct classes of IPV experience among women in India and examines how these classes are associated with their nutritional outcomes.

**Methods:**

Using data of 60,622 ever-married women aged 15–49 years from the 2019-21 National Family Health Survey (NFHS-5), latent class analysis (LCA) was performed to identify distinct groups of women based on their IPV experiences. BMI was used to assess women’s nutritional status, and it was classified as: <18.5 kg/m2 (underweight), 18.5–24.9 kg/m2 (normal) and ≥ 25.0 kg/m2 (overweight). Further, multinomial logistic regression analyses were used to estimate the odds of being underweight or overweight by latent classes of IPV experiences.

**Results:**

LCA model identified four distinct IPV experience groups of women: Low Physical and Low Sexual IPV (LPLS-IPV) class (72%); High Physical and Low Sexual IPV (HPLS-IPV) (12.5%); High Sexual and Low Physical IPV (HSLP-IPV) (12%); and High Physical and High Sexual (HPHS-IPV) class (3.5%). The likelihood of being underweight was higher among women in the HPHS-IPV class (aOR: 1.24, 95% CI: 1.08–1.44), followed by those in the HPLS-IPV class (aOR: 1.11, 95% CI: 1.04–1.20).

**Conclusion:**

The latent class groups found that high physical IPV experiences were associated with women’s nutritional outcomes. The experiences of women having both high physical and sexual violence affect women’s nutritional outcomes to a greater extent and they are more likely to be undernourished.

**Table:** 

Text box 1. Contributions to the literature	
• This is first study which uses latent class approach to identify distinct classes of intimate partner violence experiences among women in India. • The study found four diverse groups of partner violence experiences among women (a) Low Physical and Low Sexual IPV (LPLS-IPV); (b) High Physical and Low Sexual IPV (HPLS-IPV); (c) High Sexual and Low Physical IPV (HSLP-IPV); and (d) High Physical and High Sexual (HPHS-IPV). • This article found high physical partner violence experience was directly associated with women’s undernutrition.	

## Introduction

Intimate partner violence (IPV) against women, which refers to physical and coercive behaviors performed by the intimate partners (i.e., current or former husbands and live-in partners), is the manifestation of gender power [1]. According to the World Health Organization (WHO), almost one in three women globally has been subjected to physical and/or sexual violence perpetrated by an intimate partner [2]. Nonetheless, such violence does have a catastrophic impact on the health and well-being of women. Global estimates suggest that nearly 38% of all female homicides were associated with IPV [3].

Intimate partner violence against women is a public health concern in India with more than one out of four (27%) of ever-married women (aged 15–49 years) affected by IPV as per recent NFHS (2019-21) data. India has various laws, policies and programmes that seek to address gender-based violence. IPV has been declared as a criminal offence since 1983 under Indian Penal Code [4, 5]. The Protection of Women from Domestic Violence Act of 2005 (PWDVA 2005) is a civil law that defines physical, sexual, emotional, and economic abuse based on the UN Declaration on Violence Against Women and prescribes punishment as per Indian Penal Code [6]. However, there is no direct and specific state sponsored program for preventing such violence.

The survivors of IPV experience a wide range of physical and mental health consequences, including acute injuries, post-traumatic stress disorder, anxiety, damaging coping behaviors, early menopause, and sexually transmitted infections [7–10]. Several studies in India and elsewhere documented the association of IPV with increased risk of miscarriages, stillbirth, infant and child mortality, and stress and anxiety [11–14]. Research from South Asian countries and elsewhere also identified important relationships between IPV experiences and women’s nutritional outcomes. For instance, a study conducted in Bangladesh found that women exposed to physical and/or sexual IPV were at nearly 40% greater risk of being underweight than those unexposed [15]. In Nepal, women who experienced sexual IPV were 2.6 times more likely to be underweight [16]. In contrast, studies from Egypt, Nigeria, and the United States have shown that experiencing IPV was associated with increased body mass index (BMI) of women [17–19]. Thus, the existing body of research provides mixed evidence on the IPV-nutrition relationship among women. This may also point to the fact that the nutritional effects of IPV were largely context specific.

In India, malnutrition among women continues to be a pressing public health issue. The 2019-21 National Family Health Survey (NFHS) report suggested that 22% of women aged 15–49 years were underweight, whereas 14% of women in the same age group were overweight or obese [20]. Under-nutrition leads to several negative health consequences, including wasting of skeletal muscles, reduced physical work capacity, compromised immune function, and gastrointestinal disease [21, 22]. It also has severe repercussions on the reproductive health of women. For instance, underweight women of reproductive age are at an increased risk of preterm birth, low birth weight, and intra-uterine growth restriction (IUGR) [23]. On the other hand, overweight and obese individuals are particularly susceptible to many non-communicable diseases (NCDs) such as type 2 diabetes, hypertension, and pulmonary illness, which eventually inflate healthcare cost` and impair overall quality of life [24]. There have been several efforts in the last five decades through different vertical nutritional programs by the government which have improved undernutrition statistics but the decline remains slow and increasing overweight and obesity has led to double burden of nutrition.

A few studies have empirically assessed the extent to which IPV exposure predicts the risks of malnutrition among women in India [25–27]. Recent research has shown that the patterns of IPV experiences may not have a uniform impact on socioeconomic outcomes (e.g., workforce participation), rather these had a differential impact [28, 29]. In order to identify such differential impacts of IPV experience, studies have adopted person-centered approaches, such as latent class models. This approach helps researchers to identify sub-groups of women based on their exposure to various types of IPV. To date, research examining the nutritional effects of IPV on women has largely relied on the traditional categorization of IPV experiences rather than the latent class analysis (LCA) approach [23].

## Methods

### Data source

The study utilizes nationally representative data from the fifth round of the National Family Health Survey (NFHS-5) conducted during 2019-21, covering all Indian states and union territories (UTs) [20]. The NFHS-5 questionnaires collect a wide range of information from women of reproductive age 15–49, including demographic and socioeconomic characteristics, reproductive health outcomes, access to maternal health services, and anthropometric as well as biomarker measurements such as hemoglobin level, blood glucose level, blood pressure, etc. The survey protocols for NFHS-5, including the content of all the survey questionnaires, were reviewed and approved by the Institutional Review Board (IRB).

NFHS-5 adopted a two-stage sampling scheme stratified by the rural and urban samples within each state/UT. Following this procedure, a total of 699,723 households were interviewed on the national scale, with a response rate of 97.5%. Of these, a sub-sample of 15% of households was selected randomly for the implementation of a state module that collects information on additional indicators such as domestic violence, women’s empowerment, husband’s background, etc. to provide estimates at the state level. From these selected sub-samples of households, only one woman per household was selected randomly to answer questions in the domestic violence (DV) module. Thus, information regarding domestic violence was collected from 72,360 women. The present study restricts the sample to ever-married women with valid information about anthropometric measurements and other control variables (N = 60,622).

### Outcome variable

The outcome of interest, the nutritional status of women, was assessed by the Body Mass Index (BMI). In NFHS-5, anthropometric measurements of all participating women were taken by the trained health investigators. The height of women was measured using the Seca 213 Stadiometer and the weight using the Seca 874 Digital Scale. The BMI was calculated using the standard formula [weight/height^2^ (kg/m^2^)] and was classified following WHO recommended cut-off: <18.5 kg/m2 (underweight), 18.5–24.9 kg/m2 (normal) and ≥ 25.0 kg/m2 (overweight) [30].

### Exposure variables

The exposure variable used for this study is latent classes of IPV exposure. Latent classes are used to distinguish/identify unobserved homogeneous (latent) subgroups within a population [31]. The latent classes were derived using the 10 IPV indicators (7 indicators measuring physical IPV and 3 indicators measuring sexual IPV) from the DV module of NFHS-5. Regarding physical violence, each respondent was questioned about whether her (last) husband ever had: (i) “Pushed her, shaken her, or thrown something at her”, (ii) “Twisted her arm or pulled her hair”, (iii) “Slapped her”, (iv) “Punched her with his fist or with something that could hurt her”, (v) “kicked her, dragged her or beaten her up”, (vi) “tried to choke her or burn her on purpose”, (vii) “threaten or attacked her with a knife, gun, or any other weapon”. Regarding sexual IPV, each respondent was asked whether her husband ever had: (i) “Physically forced her to have sexual intercourse with him even when she did not want to”, (ii) “Physically forced her to perform any other sexual acts she did not want to”, and (iii) “Forced her with threats or in any other way to perform sexual acts she did not want to”. Responses for each of the above indicators were dichotomized as 0 (*never experienced*) and 1 (*ever experienced*) and used for the construction of latent classes of IPV.

### Control variables

Control variables for the present study were selected based on their empirical association with BMI and availability in the NFHS-5 dataset [20, 32–36]. Women’s current age (categorical variable: ‘15–24 years’, ‘25–34 years’, and ‘35–49 years’), age at marriage (‘<18 years’ vs. ‘≥18 years’), parity (categorical variable: ‘0’, ‘1’, ‘2’, and ‘3+’), women’s education (categorical variable: ‘none’, ‘primary’, ‘secondary’, and ‘higher’), working status (‘no’ vs. ‘yes’), husband’s education (categorical variable: ‘none’, ‘primary’, ‘secondary’, and ‘higher’), household wealth status (categorical variable: ‘poorest’, ‘poorer’, ‘middle’, ‘rich’, and ‘richest’), and place of residence (‘rural’ vs. ‘urban’) were included. In order to determine household’s wealth status, a wealth score was computed based on the principal component analysis (PCA) of indicators of household durable goods (e.g., TV, refrigerator, bicycle, etc.) and housing quality (e.g., type of wall, materials used for flooring, etc.), and subsequently assigned to each household. The assigned wealth score was then divided into five equal quintiles to rank the households as poorest (lowest quintile) to richest (highest quintile).

### Statistical analysis

Descriptive analysis of the study sample by demographic and socioeconomic characteristics was performed. A summary of various DV acts performed against women by their intimate partners was given.

Using responses to the 10 indicators of physical and sexual IPV described above, a series of latent class models for 2, 3, 4, and 5 classes were estimated applying maximum likelihood approach [37]. The fit of each latent class model was assessed using the Akaire Information Criterion (AIC), the Bayesian Information Criterion (BIC), and the likelihood ratio test (G2). Better model fit was determined by smaller values for AIC and BIC and statistically significant G2 statistics [38].

Further, multinomial regression models were used to assess the association of latent class membership with the nutritional outcome of women. A total of four different multinomial models were estimated. Model-1 shows a crude association between latent classes of IPV and the nutritional status of women. Model-2 controls the effects of demographic variables such as age, age at marriage, and parity. Model-3 adjusts for the effects of socioeconomic variables such as women’s education, employment status, husband’s education, household wealth, and type of residence. Finally, Model-4 controls the effects of both demographic and socioeconomic variables.

## Results

### Types of intimate partner violence experienced by women

Table 1 provides the distribution of each indicator of IPV for both physical and sexual IPV. The prevalence of any physical violence was 28.3% and any sexual violence was 6.3% among the study population. Slapping to her was the commonest form of violence reported by one-fourth (25.5%) of the women followed by pushing or shoving or throwing anything at her that could hurt (12.3%) and twisting her arm or pulling her hair (10. 2%) (Table 1). Physical force for sexual intercourse against her willingness (4.6%) and threats for sexual intercourse against her willingness (3.8%) were the most common form of sexual violence reported by Indian women.

**Table 1:** Types of violent acts performed by an intimate partner against ever-married women aged 15–49 years, India, 2019-21

Types of IPV	Types of violent acts	% (95% CI)
Physical	Pushed or shoved or thrown anything at her that could hurt	12.25 (11.99, 12.5)
	Twisted her arm or pulled her hair	10.15 (9.91, 10.38)
	Slapped her	25.25 (24.91, 25.6)
	Punched her	7.72 (7.51, 7.93)
	Kicked or dragged or beaten her up	8.27 (8.05, 8.48)
	Chocked or burnt her on purpose	2.3 (2.18, 2.41)
	Threatened or attacked her with a weapon	1.23 (1.14, 1.31)
	**Any physical**	**28.3 (27.9, 28.6)**
Sexual	Physically forced to have sexual intercourse when she did not want to	4.64 (4.47, 4.8)
	Physically forced to perform any other sexual acts when she did not want to	2.37 (2.25, 2.49)
	Forced with threats to perform sexual acts when she did not want to	3.8 (3.65, 3.95)
	**Any sexual**	**6.3 (6.0, 6.7)**

### Latent classes based on IPV Experiences

Table 2 presents fit statistics for the LCA models of the experience of IPV estimated. The four-class model had the lowest AIC and BIC values with a p-value of < 0.05 for G2 statistics. Therefore it was preferred for the final analysis.

**Table 2:** Comparisons of different LCA models

Model	AIC	BIC	logL (G2)
One-class	295692.57	295783.21	97414.80*
Two-class	216974.84	217165.19	18675.07*
Three-class	206490.81	206780.87	8169.04*
Four-class	203266.01	203655.78	4922.23*

*p < 0.05

Within the four-class model, each of the distinct classes was labeled based on the latent class probabilities (also known as item response probabilities) (Table 3). Class 1, the largest class comprising 72% of the sample, corresponded to women reportedly having low exposure to both physical and sexual violence. This group was labeled as Low Physical and Low Sexual IPV class (LPLS-IPV). Class 2, the second largest class accounting for 12.5% of the sample, comprised of women who reported a high prevalence of physical IPV and a low prevalence of sexual IPV. This class was then labeled as High Physical and Low Sexual IPV (HPLS-IPV) class. Class 3 accounted for 12% of the sample and comprised women who reported a high prevalence of sexual IPV and a low prevalence of physical IPV; this class was labeled as High Sexual and Low Physical IPV (HSLP-IPV). Class 4 is the smallest class that comprised only 3.5% of the sample; members of this group reported to have a high prevalence of both physical and sexual IPV. This class was labeled as High Physical and High Sexual (HPHS-IPV) class.

**Table 3:** Latent class probabilities of IPV

IPV item	Low physical and low sexual IPV (Class-I)	High physical and low sexual IPV (Class-II)	High sexual and low physical IPV (Class-III)	High physical and high sexual IPV (Class-IV)
Pushed	0.013 (0.012, 0.014)	0.574 (0.561, 0.587)	0.348 (0.316, 0.381)	0.924 (0.907, 0.938)
Slapped	0.099 (0.096, 0.102)	0.912 (0.903, 0.919)	0.625 (0.592, 0.657)	0.973 (0.962, 0.981)
Punched	0.003 (0.002, 0.004)	0.312 (0.300, 0.324)	0.137 (0.114, 0.163)	0.919 (0.899, 0.935)
Kicked	0.004 (0.003, 0.005)	0.331 (0.319, 0.343)	0.159 (0.136, 0.188)	0.926 (0.908, 0.941)
Chocked	0.001 (0.000, 0.001)	0.026 (0.021, 0.030)	0.048 (0.033, 0.065)	0.604 (0.576, 0.632)
Threatened	0.000 (0.000, 0.001)	0.008 (0.006, 0.011)	0.044 (0.032, 0.059)	0.390 (0.363, 0.417)
Twisted	0.003 (0.002, 0.004)	0.486 (0.473, 0.499)	0.254 (0.224, 0.286)	0.928 (0.911, 0.942)
Forced sex	0.004 (0.004, 0.005)	0.054 (0.047, 0.061)	0.627 (0.586, 0.666)	0.726 (0.696, 0.753)
Forced act	0.001 (0.001, 0.002)	0.014 (0.011, 0.018)	0.368 (0.333, 0.404)	0.539 (0.509, 0.569)
Threatened sex	0.002 (0.001, 0.002)	0.009 (0.006, 0.016)	0.673 (0.621, 0.722)	0.648 (0.618, 0.677)

### Associations between Latent Class Membership of IPV and Nutritional Status of Women

**Descriptive results.** Table 4 illustrates the prevalence of underweight and overweight among women by latent IPV classes and other demographic and socioeconomic characteristics. Result suggests that the prevalence of being underweight was highest (18.1%) among women in HPHS-IPV class, i.e., those who experienced high physical and high sexual violence, followed by those who experienced high sexual and low physical violence (HSLP-IPV) (16.5%). The prevalence of being overweight, on the other hand, was highest (29.2%) among women in LPLS-IPV class. The prevalence of being underweight was found to decline with age, whereas it was the opposite to overweight prevalence. With the increase of women’s level of education, the prevalence of underweight among women declined, but overweight prevalence increased among them. Significant differentials in the prevalence of underweight and overweight among Indian women was found by household wealth quintile groups. Nearly 23.5% of women from the poorest households were underweight compared to 5.7% of those who were from the richest households. In contrast, around 47.9% of women from the richest households were overweight compared to 12.8% from the poorest households. A significant difference in underweight and overweight prevalence was also observed by residence type.

**Table 4:** Distribution of women’s BMI categories by IPV latent class, demographic, and socioeconomic characteristics, India, 2019-21

	% (95% CI) of BMI Categories		
Variable	Normal	Underweight	Overweight
IPV Latent Class			
LPLS	57.4 (56.9, 57.8)	13.4 (13.1, 13.7)	29.2 (28.8, 29.6)
HPLS	59.5 (58.4, 60.6)	15.1 (14.3, 15.9)	25.5 (24.5, 26.4)
HSLP	60.7 (57.9, 63.5)	16.5 (14.4, 18.7)	22.8 (20.4, 25.2)
HPHS	57.2 (54.8, 59.5)	18.1 (16.3, 19.9)	24.8 (22.7, 26.8)
**Age Group (Years)**			
15–24	63.8 (62.8, 64.8)	23.2 (22.4, 24.1)	13.0 (12.3, 13.7)
25–34	59.1 (58.4, 59.7)	15.0 (14.5, 15.5)	25.9 (25.4, 26.5)
35–49	54.9 (54.3, 55.5)	10.1 (9.8, 10.4)	35.0 (34.5, 35.6)
**Age at Marriage (Years)**			
< 18	58.4 (57.8, 59.0)	14.9 (14.5, 15.4)	26.6 (26.1, 27.2)
≥ 18	57.0 (56.5, 57.6)	12.8 (12.4, 13.2)	30.2 (29.7, 30.7)
**Parity**			
0	61.8 (60.5, 63.2)	14.9 (13.9, 15.9)	23.2 (22.1, 24.4)
1	59.5 (58.6, 60.4)	15.0 (14.3, 15.7)	25.5 (24.7, 26.3)
2	54.7 (54.1, 55.4)	12.1 (11.7, 12.5)	33.2 (32.5, 33.8)
3+	58.7 (58.1, 59.4)	14.6 (14.1, 15.0)	26.7 (26.1, 27.3)
**Woman’s Education**			
No schooling	61.0 (60.3, 61.7)	17.1 (16.5, 17.6)	21.9 (21.3, 22.5)
Below primary	59.8 (58.7, 60.8)	14.2 (13.5, 14.9)	26.0 (25.1, 26.9)
Secondary	55.6 (55.0, 56.2)	13.1 (12.7, 13.5)	31.3 (30.8, 31.8)
Higher	55.1 (53.9, 56.4)	7.6 (7.0, 8.3)	37.2 (36.0, 38.4)
**Current Working Status**			
Not working	57.6 (57.2, 58.1)	13.4 (13.1, 13.7)	29.0 (28.5, 29.4)
Working	57.9 (57.2, 58.6)	14.7 (14.2, 15.2)	27.4 (26.8, 28.1)
**Husband’s Education**			
No schooling	61.2 (60.3, 62.1)	17.7 (17.0, 18.4)	21.1 (20.4, 21.9)
Below primary	59.4 (58.4, 60.4)	16.1 (15.3, 16.8)	24.5 (23.6, 25.4)
Secondary	56.9 (56.4, 57.5)	13.2 (12.8, 13.5)	29.9 (29.4, 30.4)
Higher	53.4 (52.3, 54.5)	8.2 (7.6, 8.7)	38.4 (37.4, 39.5)
**Household Wealth Status**			
Poorest	63.7 (62.8, 64.6)	23.5 (22.7, 24.3)	12.8 (12.2, 13.4)
Poorer	63.3 (62.4, 64.1)	16.9 (16.2, 17.5)	19.8 (19.2, 20.5)
Middle	58.4 (57.5, 59.2)	13.6 (13.0, 14.2)	28.0 (27.2, 28.8)
Richer	54.6 (53.7, 55.5)	8.1 (7.6, 8.6)	37.3 (36.5, 38.2)
Richest	46.4 (45.4, 47.4)	5.7 (5.2, 6.1)	47.9 (46.9, 48.9)
**Type of Residence**			
Urban	51.8 (51.0, 52.5)	7.9 (7.5, 8.3)	40.3 (39.6, 41.0)
Rural	60.2 (59.7, 60.7)	16.3 (15.9, 16.6)	23.5 (23.1, 23.9)

IPV = Intimate Partner Violence; LPLS = Low Physical and Low Sexual; HPLS = High Physical Low Sexual; HSLP = High Sexual and Low Physical; HPHS = High Physical and High Sexual

**Results from multinomial logistic regression.** In line with descriptive results presented above, the latent classes of IPV experiences show a profound association with the nutritional status of women (Table 5). The unadjusted analysis (Model-1) suggests that the risk of being underweight was highest among women in HPHS-IPV class (OR: 1.38, 95% CI: 1.20–1.58). Women belonging to HPLS-IPV class (OR: 1.25, 95% CI: 1.17–1.34) and HSLP-IPV class (OR: 1.24, 95% CI: 1.06–1.46) also had increased odds of being underweight, in comparison with LPLS-IPV class.

**Table 5:** Multinomial logistic regression showing odds ratio for of BMI categories for IPV latent class groups

IPV class membership	Underweight vs. Normal		Overweight vs. Normal
	OR (95% CI)		OR (95% CI)
***Model − 1***			
Low physical & low sexual (LPLS)	ref.		ref.
High physical & low sexual (HPLS)	1.25 (1.17, 1.34)		0.82 (0.77, 0.86)
High sexual & low physical (HSLP)	1.24 (1.06, 1.46)		0.68 (0.58, 0.79)
High physical & high sexual (HPHS)	1.38 (1.20, 1.58)		0.88 (0.78, 1.00)
***Model − 2***			
Low physical & low sexual (LPLS)	ref.		ref.
High physical & low sexual (HPLS)	1.24 (1.16, 1.33)		0.81 (0.77, 0.87)
High sexual & low physical (HSLP)	1.21 (1.02, 1.43)		0.70 (0.60, 0.82)
High physical & high sexual (HPHS)	1.40 (1.22, 1.62)		0.88 (0.77, 0.99)
***Model − 3***			
Low physical & low sexual (LPLS)	ref.		ref.
High physical & low sexual (HPLS)	1.11 (1.03, 1.18)		1.01 (0.95, 1.07)
High sexual & low physical (HSLP)	1.13 (0.96, 1.33)		0.79 (0.67, 0.92)
High physical & high sexual (HPHS)	1.20 (1.04, 1.39)		1.10 (0.97, 1.25)
***Model − 4***			
Low physical & low sexual (LPLS)	ref.		ref.
High physical & low sexual (HPLS)	1.11 (1.04, 1.20)		0.98 (0.92, 1.04)
High sexual & low physical (HSLP)	1.11 (0.94, 1.31)		0.80 (0.68, 0.94)
High physical & high sexual (HPHS)	1.24 (1.08, 1.44)		1.05 (0.92, 1.20)

After adjusting for demographic characteristics (Model-2), the strength of associations between IPV class membership and the likelihood of being underweight declined slightly. Adjustment for socioeconomic variables (Model-3) led to further attenuation in the strength of associations. Of note, the odds ratio of the HSLP-IPV class (OR: 1.13, 95% CI: 0.96–1.33) was no longer statistically significant.

Finally, after controlling both demographic and socioeconomic confounders (Model-4), women in HPLS-IPV (OR: 1.24, 95% CI: 1.08–1.44) and HPHS-IPV (OR: 1.11, 95% CI: 1.04–1.20) classes remained significantly associated with lower odds of being underweight.

## Discussion

Studies have widely documented how various biological, demographic, and socioeconomic risk factors contribute to malnutrition [32, 33, 39, 40]. However, the impact of violence on women’s nutrition is not widely studied in the Indian context. The current study examined the nutritional risks associated with intimate partner violence—a psychosocial factor to which nearly one-third of adult Indian women are exposed. Using latent class analysis, the study first identified the typologies of IPV experiences among women and then examined how these typologies are associated with nutritional outcomes among Indian women.

Four distinct IPV experience groups (i.e., latent classes) were identified in this study. These distinct latent classes of IPV appeared to be differently associated with nutritional outcomes. The likelihood of being underweight was greatest among women in the HPHS-IPV class, which constituted approximately 3.5% of the total sample. Women in the HPLS-IPV class also had a greater risk of being underweight in comparison to the LPLS-IPV class. Notably, HSLP-IPV class did not significantly relate to higher odds of being underweight, especially when socioeconomic factors were taken into account. It is very likely that the overriding effect of certain socioeconomic variables (e.g., household wealth) on nutritional outcomes has led to a weakened relationship between this class and nutritional outcomes. It may be that the detrimental health consequences of belonging to HSLP-IPV class may exhibit other health outcomes, namely psychological or reproductive health [26, 41].

Prior research investigating the typologies of IPV against women in India has mostly employed binary indicators to measure an individual’s exposure to IPV. Limited studies have used the LCA approach to investigate the typologies of IPV against women in India, and therefore direct comparisons of the distinct classes identified in this study are challenging. Nonetheless, findings of the present study are well-aligned with prior research that underscores how the identification of distinct IPV classes improves the understanding of the heterogeneous nature of violence that the vulnerable group experience [28, 42–44].

The association that has been observed in the present study between HPHS-IPV and being underweight corroborates the prior studies conducted in India and other similar contexts. Ackerson and Subramanian [25], for instance, reported that women who had undergone physical IPV (anytime in the last 12 months) were 1.38 times more likely to be underweight. In Bangladesh, Rahman et al. [15] reported that exposure to physical and/or sexual IPV was significantly positively associated with women’s lower BMI. Despite being consistent, the direct comparison of the results of prior studies with the present study is challenging due to two reasons. Firstly, the membership of IPV classes in the present study was determined based on latent probability, whereas the prior studies [25, 40] relied on manual classification of IPV types; such methodological differences, that may yield varied results. Secondly, these studies used a different set of confounders which may also influence the results. Household socioeconomic status, for instance, was not adjusted for in the Bangladesh study [15], whereas, the present study found household wealth to be a significant confounder in the analysis.

## Conclusion

This population-based study in India identified four distinct IPV experience groups of women. The latent class groups found that high physical IPV experiences were associated with women’s nutritional outcomes. The experiences of women having physical violence also affect their nutritional outcomes to a greater extent and they are more likely to be undernourished. Overall, the present study’s findings suggest that strategies to eliminate IPV against women are important to address malnutrition among women in India.

### Implications to Research and policies

The findings from the study suggest that high physical violence is associated with undernutrition of women. There is need for further research on pathways that impact woman nutrition due to physical violence. Nevertheless, the evidence call for the need for framing a policy to prevent any form of physical violence for better nutrition and gender equity. There is a need for trained counselors at the community level to provide marital counseling for stained marital relationships and prevention of violence against women.

### Strengths and Limitations

The present study has several strengths. The application of LCA model—which is a person-centered modelling approach that estimates an individual’s probability of membership to select variables in a specific class. This model has allowed this study to create most appropriate IPV groups and detect meaningful differences in nutrition outcomes among the IPV groups. Further, the use of a nationally representative large-scale survey data enhanced the statistical power in the analyses of the study. These strengths notwithstanding, the findings of the study should be interpreted in the light of a few limitations. Firstly, the latent classes were constructed based on women’s self-reported experience of IPV [41, 45, 46]. Underreporting of cases may influence latent classes, and thereby impacting the association between the outcome and the latent classes. Secondly, the latent classes were derived from a select set of indicators (i.e., IPV items). Analyses incorporating different set of IPV indicators or indicators related to the experience of IPV (e.g., injuries following IPV) may yield different latent classes. Therefore, the generalization of latent classes should be made carefully. It may also be noted that the data collectors for NFHS are well-trained for longer durations and were trained to maintain privacy and confidentiality of the respondents in all circumstances. Finally, the cross-sectional design of the present study has restricted it to draw any causal inferences regarding the associations.

## Data Availability

Data for the 2019-21 NFHS is publicly available with no individual identifiers. It is freely accessible from https://dhsprogram.com/.
